# A Computational Theory for the Emergence of Grammatical Categories in Cortical Dynamics

**DOI:** 10.3389/fncir.2020.00012

**Published:** 2020-04-16

**Authors:** Dario Dematties, Silvio Rizzi, George K. Thiruvathukal, Mauricio David Pérez, Alejandro Wainselboim, B. Silvano Zanutto

**Affiliations:** ^1^Universidad de Buenos Aires, Facultad de Ingeniería, Instituto de Ingeniería Biomédica, Buenos Aires, Argentina; ^2^Argonne National Laboratory, Lemont, IL, United States; ^3^Computer Science Department, Loyola University Chicago, Chicago, IL, United States; ^4^Microwaves in Medical Engineering Group, Division of Solid-State Electronics, Department of Electrical Engineering, Uppsala University, Uppsala, Sweden; ^5^Centro Científico Tecnológico Conicet Mendoza, Instituto de Ciencias Humanas, Sociales y Ambientales, Mendoza, Argentina; ^6^Instituto de Biología y Medicina Experimental-CONICET, Buenos Aires, Argentina

**Keywords:** cortical dynamics, grammar emergence, brain-inspired artificial neural networks, unsupervised learning, computational linguistics, online sentence processing

## Abstract

A general agreement in psycholinguistics claims that syntax and meaning are unified precisely and very quickly during online sentence processing. Although several theories have advanced arguments regarding the neurocomputational bases of this phenomenon, we argue that these theories could potentially benefit by including neurophysiological data concerning cortical dynamics constraints in brain tissue. In addition, some theories promote the integration of complex optimization methods in neural tissue. In this paper we attempt to fill these gaps introducing a computational model inspired in the dynamics of cortical tissue. In our modeling approach, proximal afferent dendrites produce stochastic cellular activations, while distal dendritic branches–on the other hand–contribute independently to somatic depolarization by means of dendritic spikes, and finally, prediction failures produce massive firing events preventing formation of sparse distributed representations. The model presented in this paper combines semantic and coarse-grained syntactic constraints for each word in a sentence context until grammatically related word function discrimination emerges spontaneously by the sole correlation of lexical information from different sources without applying complex optimization methods. By means of support vector machine techniques, we show that the sparse activation features returned by our approach are well suited—bootstrapping from the features returned by Word Embedding mechanisms—to accomplish grammatical function classification of individual words in a sentence. In this way we develop a biologically guided computational explanation for linguistically relevant unification processes in cortex which connects psycholinguistics to neurobiological accounts of language. We also claim that the computational hypotheses established in this research could foster future work on biologically-inspired learning algorithms for natural language processing applications.

## 1. Introduction

Given the complexity of human language, it is difficult to understand how children can exploit its internal structure in order to convey meaningful communicative behavior. Nevertheless, most of them achieve such behavior successfully within the first few years of life (Saffran et al., 2001). Some lines of research highlight the importance of the statistical structure underlying language in general (Romberg and Saffran, 2010; Lopopolo et al., 2017), while others show that 11–20 month-olds are able to acquire different aspects of abstract grammatical rules (Cyr and Shi, 2013; van Heugten and Christophe, 2015).

Many psycho-linguistic models propose that in on-line sentence processing, different types of constraints are integrated very quickly in a coherent manner determining how words are systemically combined in grammatical sentences (Gibson and Pearlmutter, 1998). It is proposed that qualitatively distinct constraints such as semantic/conceptual, phonological, and syntactic structures operate alongside on a referential binding into a discourse model (Rego and Bryant, 1993; Lopopolo et al., 2017). In some models, unification operations during sentence comprehension take place in a parallel fashion at the semantic, syntactic, and phonological levels of processing (Hagoort, 2005). During on-line comprehension, lexical items are processed sequentially as the time course of the input elapses. The structural frames associated with each word are combined by means of an incremental unification mechanism, in the order that the input imposes.

In the present work, we introduce a bio-inspired neurocomputational model in which each word from the mental lexicon is associated with a structural frame. Each structural frame consists of the combination of Distributional Semantic (DS) (Harris, 1954) and coarse-grained syntactical word category information—specifically *function word* category, *content word* category, and from the last one we segregate *verb word* category. The coarse-grained word category information used in this work has been shown to emerge from phonological constraints in early language acquisition (Shi et al., 2006; Lohmann, 2017). A structural frame used in this approach constitutes the environment for a particular lexical item. In this model, constituent structures are established by an unification operation which consists of linking up DSs, phonologically grounded coarse syntax and sequential constraints correlating them repeatedly until constituent grammatical classification improvement spontaneously emerges. Classification improvement emergence in grammatical relevant information is obtained without any kind of optimization guidance beyond the correlation of the different constraints.

In psycho-linguistics it is proposed that only one phrasal configuration remains active among the alternative binding candidates. Such selection mechanism would be achieved by means of a lateral inhibition process between two or more alternative unification links (Hagoort, 2005). In the same way, in our neurocomputational model, information coming from lateral and apical dendrites constrains the massive activation of units excited by afferent dendrites (Dematties et al., 2019). Afferent dendrites receive Distributional Semantic (DS) constraints while apical dendrites receive coarse-grained syntactical constraints.

**Figure 1:**
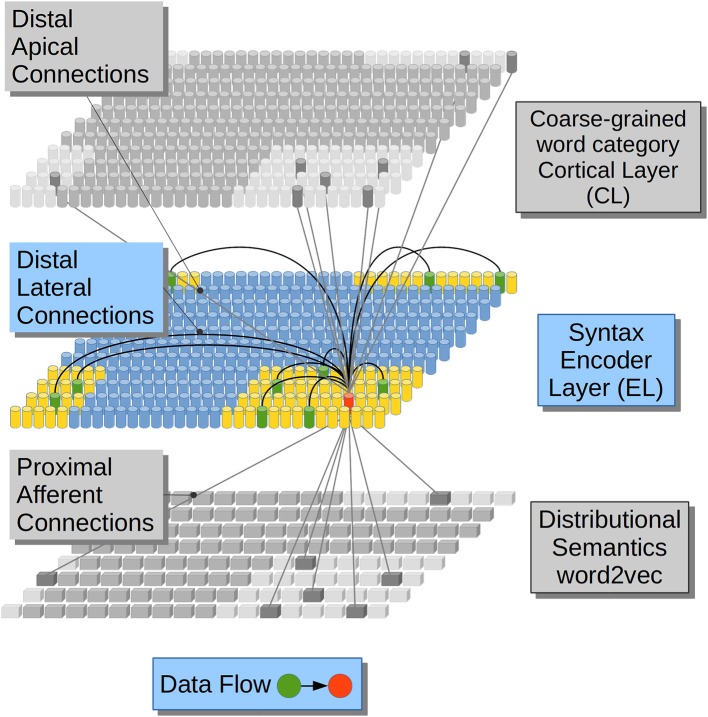
Computational hypotheses. Connection scheme for a Cortical Column (CC) in the Encoder Layer (EL). Each cylinder in the EL and in the Cortical Layer (CL) represents a CC in neural tissue. Each prism in Distributional Semantics (word2vec) represents a real-valued variable. This is a visualization of a CC (in red) and its three receptive fields (lateral in yellow and afferent and apical in light gray). Adapted from https://doi.org/10.1371/journal.pone.0217966 under CC-BY license.

In regards to the emergence of coarse-grained syntactical constraints, phonologically-based implicit-learning mechanisms have been shown to serve as a precursor to later grammar learning in 4-month-old infants (Friederici et al., 2011), in such sense phonology serves the recognition and representation of *function words* in English-Learning infants (Shi et al., 2006), and in the derivation process between *nouns* and *verbs* in English (Lohmann, 2017). Lateral dendrites, on the other hand, receive information from the previous activations in the same cortical patch making the network aware of the sequence of lexical constituents along each sentence (Figure 1).

In our computational model DS constraints from afferent dendrites excite clusters of neurons in the Encoder Layer (EL) stage in Figure 1. The EL may be related to a cortical patch composed by the Brodmann Area (BA) 45 and the Brodmann Area (BA) 44 which are believed to contribute to syntactic processing (Friederici, 2011; Pallier et al., 2011; Hagoort and Indefrey, 2014). The EL receives Distributional Semantic (DS) constraints in its afferent dendrites. This simulation feature accounts for information coming from (BA) 47 and 45 which are involved in semantic processing (Zhang et al., 2004; Carli et al., 2007; Newman et al., 2010; Goucha and Friederici, 2015). DSs information tends to activate clusters of units in the EL. Such activations are massive at first, covering all the plausible lexical hypotheses that the Distributional Semantic (DS) information conveys. All the lexical hypotheses activated by afferent dendrites are narrowed down by distal dendrites receiving previous activations from the very same EL (lateral) and by distal dendrites receiving coarse-grained word category information (apical) which could be related to BA 44 and part of BA 6 which have a role in phonological processing (Heim et al., 2003, 2008; Amunts et al., 2004; Lee et al., 2012; Lima et al., 2016). Distal connections partially depolarize specific neural units in the EL which will get a running start on their activations compared to neighboring units, when afferent information arrives. Partially depolarized units will activate faster than their counterparts, inhibiting their depolarization and, in this way, preventing them from firing. By means of such strategy, the EL generates Sparse Distributed Representations (SDRs) with a 99% of sparsity, popping up only one choice among all the alternative unification links. We propose that in such way only one phrasal configuration remains active among the alternative binding candidates.

Apical dendrites receive feedback connections (Petro et al., 2014; Phillips et al., 2018; Eberhardt et al., 2019) from higher-order to lower-order cortical areas, often related to attention, prediction, expectation, and awareness (Marques et al., 2018). The role of such connections is usually related to modulatory functions which control the responsiveness to more proximal inputs and guide the effects produced by forward connections (Spruston, 2008; Chen et al., 2009; Marques et al., 2018). In our current implementation, we assign coarse-grained syntax information to the simulation of apical dendrites in the model. With this decision we do not claim that real apical trees are confined to this role; rather, this feature is adopted since it was the most suitable one for the model in its current state of development (see further details in section 2). Hence we assign DS information a forward-driving role in our model which is modulated by previously activated information from distal (apical and lateral) dendrites. This implies that at its current state, and due to implementation reasons, processing of syntax information precedes semantic information in the model. Nevertheless, there is ample experimental evidence that puts semantic processes as early in time as syntactic ones (Egorova et al., 2013; Moseley et al., 2013). Even more recent neurophysiological results indicates that semantic information present *before* arrival of the linguistic stimulus actually leads to different semantic predictions, demonstrating that semantic processes happen concurrently with syntactic ones (Grisoni et al., 2019). Future and more complex implementations of the model will take this evidence into account.

Thus, in the present work, we introduce a fully unsupervised approach in which constraints from different sources are correlated repeatedly until grammatically related word category generalization naturally emerges from the statistical properties of the stimulus. Our computational model does not apply any form of optimization guidance other than the gradual decrease of the learning rate. It does not backpropagate errors, nor optimize weights based on hypothetical cost functions.

An influential trend of compelling research is currently trying to explain how Back-Propagation might be carried out by neural tissue in the brain (Whittington and Bogacz, 2019). Biologically questionable aspects of the Back-Propagation algorithm such as the lack of local error representation and the need of symmetry in forward and backward weights are addressed by sound neurocomputational theories (Lillicrap et al., 2016; Guerguiev et al., 2017). Although such theories contribute with powerful arguments favoring the fact that Back-Propagation could have been implemented by evolution in cortical tissue, empirical evidence is far from conclusive. We believe there is still a long way to go before we can assure that the complex requirements imposed by *credit assignment*—the ultimate goal of Back-Propagation—could be a phenomenon occurring in cortical tissue. We are also skeptical regarding Backpropagation Through Time (BPTT) mechanisms in cortex given the demanding requirements they impose in its implementation. BPTT has particular problems specifically regarded to Temporal Credit Assignment (TCA) which are not present in feedforward backpropagation (Lillicrap and Santoro, 2019).

In such regard, we remain cautious, keeping our model as simple as possible. We do not implement reinforcement mechanisms either. Instead, we feature strong evidence from current deep neural network approaches in which spontaneous emergence of abstract concepts seems to be a new landmark for Machine Learning (ML). For instance, it has been seen that biologically-inspired deep neural networks spontaneously gain number sense even when trained only to recognize objects in an image (Nasr et al., 2019). Moreover, using the same computational hypotheses than in the present work, our group has shown how phonetic invariance and generalization spontaneously emerges from the sequential constraints imposed by the statistical structure of the stimulus. Avoiding the utilization of optimization guidance mechanisms—such as supervision or reinforcement—we could significantly improve the Support Vector Machine (SVM) phonetic classification performance of stimuli seriously impaired by different levels of white noise, reverberation and changes in pitch and voices (Dematties et al., 2019).

Unlike our previous work devoted to the analysis of cortical activation for invariance in the phonetic classification of words, in this research we address grammar emergence. In this paper we approach the interaction of information from extra- and coarse-sources injected to apical dendrites in addition to the information delivered by proximal synapses and the sequential information provided by distal lateral dendrites considered in our previous work. Proximal synapses are fed with DSs in the present research. In contrast, proximal synapses in Dematties et al. (2019) were fed with phonetic features delivered by a Multiresolution Spectro-Temporal Sound Analysis (MRSTSA) algorithm inspired in Chi et al. (2005). The number of tags in the classification challenge faced by the present research substantially exceeds the one presented in the previous work. In fact, in the current presentation we used the assistance provided by additional tools—such as a natural language parser for English—in order to obtain the grammatical categories for each sentence in the corpora. We also conducted segregated analyses inspecting the classification performance for each grammatical tag and clustering the grammatically related categories in convenient subgroups. We conducted the same performance tests for an instance of the model without distal lateral connectivity. With such experiments we could determine the contributions that each dendritic compartment renders to the grammar learning task. Finally, in the current research we also analyzed the probabilistic prediction values returned by the classification algorithms for the analysis of the constituents inside a single sentence.

In the present work, we advance an improved version of the neurocomputational model previously developed in Dematties et al. (2019). In its present form, afferent dendrites drive Distributional Semantic (DS) Text Embedding information, while lateral dendrites receive sequential syntactic restrictions but, more importantly, we incorporate apical dendrites which simulate backward connectivity from distant cortical patches carrying coarse-grained word category information which has been shown to be phonologically informed (Shi et al., 2006; Lohmann, 2017). Backward connectivity has been seen to be prevalent in brain cortex, usually related to modulatory functions, driving effects produced by forward connections, and transcending more than one cortical level (Chen et al., 2009; Marques et al., 2018; News, 2018). Therefore, we show how our model—specifically the EL—in its current form, displays the acquisition of complex cognitive phenomena such as grammatically relevant categories, improving the SVM classification of grammatical functions within a sentence, compared to current word embedding representations (Mikolov et al., 2013a,b,c).

In this paper we researched and included some features of the Left Inferior Frontal Gyrus (LIFG) information processing gradient in order to settle the stream of linguistic information in our model. Modeling the entire neurophysiology present in the LIFG complex is beyond the scope of this research. We impose biological constraints to such information processing stream by means of general—and widely acknowledged—biological claims by basing our reasoning on the homogeneity found throughout cortical tissue in the brain (Carlo and Stevens, 2013).

## 2. Materials and Methods

### 2.1. Computational Model

We propose a computational approach inspired in the biology of the mammalian neocortex which simulates a patch of cortical tissue and incorporates columnar organization, spontaneous micro-columnar formation, Sparse Distributed Representation (SDR) activations which have shown to be derived from partial N-Methyl-D-aspartic acid (NMDA) dendritic depolarization (Antic et al., 2010; Major et al., 2013; Hawkins and Ahmad, 2016), and adaptation to contextual activations. We simulate pyramidal cells with proximal connections from afferent dendritic branches and distal connections from lateral and apical dendritic branches (Dematties et al., 2019).

In our model we account for layer II/III pyramidal cells processing and the segregation of their dendritic trees into different confined domains. Different dendritic domains in the cell receive distinct synaptic inputs and have synapses with specific excitability, modulation and plasticity properties (Spruston, 2008). The basal and proximal apical dendrites of cells in layer II/III take inputs from layer IV cells and also receive local-circuit excitation which process excitatory inputs from local sources. The apical tuft—on the other hand—collects inputs from other cortical areas and also receives nonspecific thalamic inputs which control responsiveness to more proximal inputs (Larkum et al., 2004).

Regarding proximal afferent connectivity in the EL, evidence shows that layer II/III predominately accepts inter-laminar—mostly vertically oriented—excitatory inputs from the stellate cells in layer IV. On the other hand the use of lateral distal connectivity accounts for layer II/III neurons which also receive excitatory inputs from other layer II/III neurons through extensive lateral connections (Bannister, 2005). Finally, and as an output gateway, many layer II/III neurons project to higher levels of cortex (Thomson and Bannister, 1998).

Hence the information from distal dendritic branches in our model—which is lateral and apical—produces, in advance, a partial depolarization in some cell units in a CC (Figure 1). On the other hand, information from proximal dendritic branches—which is afferent—produces a complete depolarization of a cluster of cell units in a Cortical Column (CC), but in the event that enough afferently excited units have already been partially depolarized by lateral and/or apical dendrites, such units would fire before inhibiting other units in the cluster and preventing them from firing. With this mechanism, only a reduced number of units become active, producing a sparse pattern of activation in our model. This neurocomputational theory has been introduced in Hawkins and Ahmad (2016), showing continuous online and unsupervised sequence learning capabilities (Cui et al., 2016).

Information processing in layer II/III concerns *sequential state inference* which requires the interaction of their lateral and apical connections with the bottom-up input. Evidence suggest that neurons in layer II/III could endeavor similar computations to the one developed by our model. Yoshimura et al. (2000) reported that long distance horizontal connections to pyramidal cells in layer II/III exhibit different properties than those from vertical connections. Feldmeyer et al. (2002) also suggested that the projections of layer IV spiny neurons to layer II/III pyramidal neurons act as a gate for the lateral spread of excitation in layer II/III. Based on such evidence we assign DS a driving role which is modulated by previous syntax activations from apical dendrites and previous lateral constraints generated in the same EL.

Some important remarks in reference to our computational approach (Dematties et al., 2019) are: (i) proximal afferent dendrites do not determine a neuron to fire, instead, they bias its probability of doing so, (ii) distal dendritic branches are independent computing elements that contribute to somatic firing by means of dendritic spikes, and (iii) prediction failures in the network produce a phenomenon called Massive Firing Event (MFE) which manifests with the activation of many neurons in a CC impairing SDRs formation.

In reference to the random nature imprinted in the computational approach, previous studies have already incorporated stochastic forces to biologically plausible models of neuronal dynamics (Harrison et al., 2005). In addition, the autonomy of neural dendrites as independent elements of computation has already been posed, showing that neuronal dendrites exhibit a range of linear and non-linear mechanisms that allow them to implement elementary computations (Poirazi, 2015; Payeur et al., 2019). The compartmentalization of individual (layer II/III) neural units in our model is not limited to only complete dendritic configurations; we are rather motivated by Jadi et al. (2014) who suggested that complex interaction between inputs delivered to two different dendritic sites could produce—for instance—exclusive OR computations. Furthermore, recent scientific studies claim that individual dendritic compartments can perform a specific computation—exclusive OR—that mathematicians had previously regarded as an unsolvable problem by single-neuron systems (Gidon et al., 2020). Finally MFEs in the model explain integration phenomena in which a combination of different constraints converges incoherently and produces the massive activation of a neuron cluster in the CCs of the Encoder Layer (EL). When the EL cannot fluently integrate information coming from different linguistic constraints, it activates more hypotheses—i.e., more phrasal configurations—as to be able to easily fuse subsequent information coming within the sequential sentence context.

An activation phenomenon such as the Massive Firing Event (MFE) impedes SDRs formation. However,when the EL correctly predicts the sequential stream of information coming from different constraints, it continuously produces SDRs and the sequential activation runs smoothly. SDRs exhibit interesting mathematical properties which give them high noise rejection and fault tolerance (Ahmad and Hawkins, 2015). It has been shown that the brain uses sparse patterns of activation to process information in all mammals, from mice to humans (Barth and Poulet, 2012).

### 2.2. Afferent Distributional Semantic Constraints

We generate Distributional Semantic (DS) constraints using Word Embedding approaches. Word Embedding is a set of Natural Language Processing (NLP) techniques in which words or phrases from a vocabulary are mapped to vectors of real numbers. We specifically use word2vec which takes a large corpus of text as input and produces a vector space that usually has several hundred dimensions. Each word in the corpus is assigned to a corresponding vector in the space. The main hypothesis is that words which recurrently appear in proximal positions in the text will be located proximally in the semantic space (DS vector space). The hypothesis is based on Distributional Semantic (DS) in linguistics which is derived from the semantic theory of language usage (Harris, 1954). The output of such model is a semantic multi-dimensional space with compelling semantic properties (Mikolov et al., 2013a,b,c). In this paper we used pre-trained vectors obtained from part of Google News dataset (about 100 billion words). The model contains 300-dimensional vectors for 3 million words and phrases (Google, 2019).

The major excitatory projection received by layer II/III neurons is mostly vertically-oriented and, for the most part, intra-columnar axons from layer IV neurons (Lübke and Feldmeyer, 2007; Thomson and Lamy, 2007). Layer IV is generally accepted as the feed forward input layer to cortical regions (Hegdé and Felleman, 2007). Consequently we interpret such microcircuit feature as an input gateway from which layer II/III neurons get proximal afferent excitation from Distributional Semantic DS.

**Figure 2:**
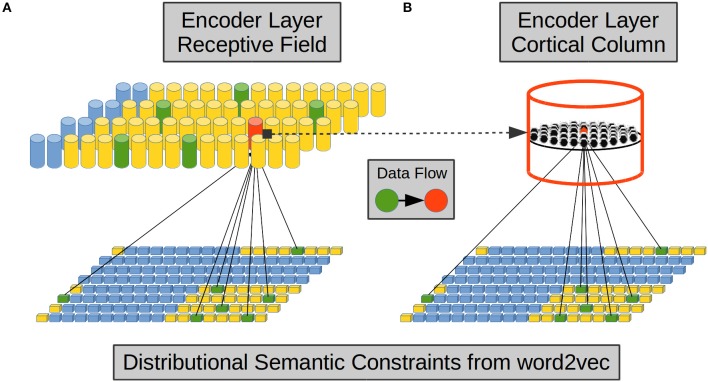
Encoder Layer (EL) proximal afferent connections. Each CC in the EL—exemplified here in red—has its receptive field over the word2vec semantic space—in yellow. **(A)** A set of word2vec components—in green inside the receptive field—is randomly chosen to be connected with a CC. **(B)** Each neural unit in a CC is connected with the same set of word2vec components. We based this connectivity configuration on the vertical-orientation and on the prominent intra-columnar configuration of afferent axons received from layer IV. Adapted from https://doi.org/10.1371/journal.pone.0217966 under CC-BY license.

From the above evidence we implemented afferent dendrites by means of Self Organizing Maps (SOMs) (Kohonen, 1988, 1989). Each CC in the EL simulates one proximal afferent dendrite using a SOM as shown in Figures 2A,B. Each CC receives a reduced sample from the word2vec components.

The use of a SOM per CC in our model accounts for proximal lateral intra-column interaction, Long-Term Potentiation (LTP), Long-Term Depression (LTD), and the vertical organization of cell bands with similar response properties (Mountcastle et al., 1955; Haueis, 2016). In each CC in our model, clusters of neural units responding to similar semantic constraints emerge spontaneously from learning. This has a correlate with the *tonotopic, retinotopic*, or *somatotopic* organization in brain tissue. In our modeling approach we account for the concept of cortical column as an *elementary unit of organization* of the entire cerebral cortex (Mountcastle, 1957, 1978, 1997). Each cortical column consists of a Self Organizing Map (SOM) which lumps neurons together arranging them in mini-columns. A mini-column denotes a thin vertical arrangement of neurons (Buxhoeveden and Casanova, 2002) and cells within a mini-column recognize the same feedforward patterns (Jones, 2000). The terms mini-column and micro-column are used interchangeably in the works by Jones (2000) and Buxhoeveden and Casanova (2002). In the present work are therefore considered synonyms.

**Figure 3:**
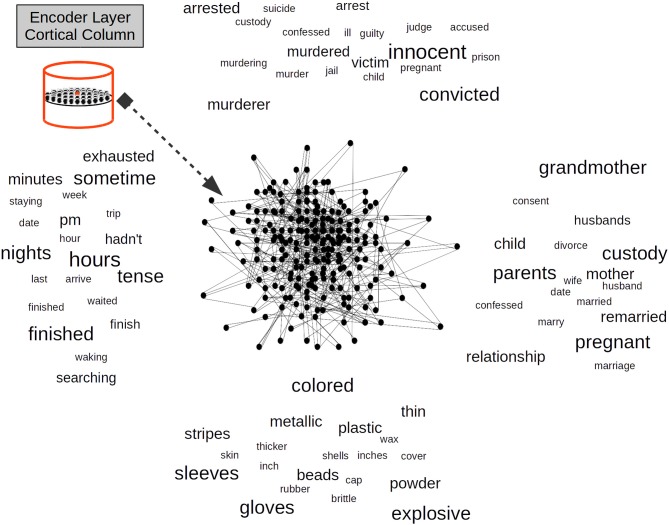
Encoder Layer (EL) Cortical Column (CC) and its proximal afferent dendrite whose synapses are simulated by a Self Organizing Map (SOM). Each CC in the EL—exemplified here in red—has its receptive field over word2vec as a semantic sub-space represented by words scattered in the background. SOM weights are in their initial state. Adapted from https://doi.org/10.1371/journal.pone.0217966 under CC-BY license.

**Figure 4:**
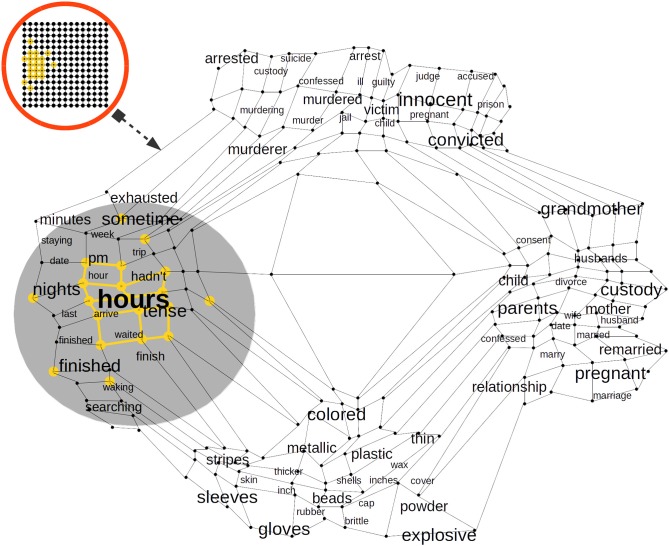
A trained Self Organizing Map (SOM) in a Cortical Column (CC) proximal afferent connections. The SOM is a bi-dimensional lattice of 225 neural units (15 × 15 neural units). The SOM adapts to represent the word2vec Distributional Semantic (DS) sub-space. After it is trained, a SOM in a CC distributes its neural units throughout the DS sub-space sampled from word2vec. The SOM algorithm keeps the semantic topology of the original DS sub-space imprinted in the lattice of neural units. Each word in the DS sub-space has its neural representation and words with more semantic similarity are represented by neural units with a high physical proximity in the lattice.

Figures 3, 4 show the relationship between the word2vec DS space and each CC in the EL. In Figure 3, the CC afferent dendrite is in its initial state and its corresponding semantic sub-space sampled from word2vec is represented by several words scattered in the background.

Each set of afferent connections in a CC determines a two-dimensional lattice of 15 × 15 neural units. The sampled semantic sub-space has 31 real valued components. In Figure 4, once the CC afferent dendrite has been trained—the neural lattice distributes its neural units throughout the semantic sub-space. In such case, each word in the semantic sub-space will have its neural resource representation and words with semantic proximity in the semantic sub-space will be represented by neural resources with physical proximity in the lattice. The neighborhood preservation of self-organizing feature maps is an important property which turns out to be useful to preserve the original sub-space semantic relationships in the new space in the lattice of a CC in the cortex (Villmann et al., 1997). Yet, even more importantly, the DS remapping from word2vec subspace to the reduced dimensionality of the neural lattice reveals hidden relationships among the terms in the original sub-space as is the case in Latent Semantic Analysis (LSA) (Boling and Das, 2015).

Once afferent dendrites have learned the statistical distribution immersed in the different Distributional Semantic (DS) sub-spaces, each CC in the EL has its private representation of its DS sub-space from word2vec. The advent of the DS representation from certain word in word2vec establishes a pattern of activation in a cluster of neural units inside each CC (Figure 4). In this way, each DS constraint from word2vec will be represented in a distributed way throughout the EL. The semantic representation of each word will activate a cluster of neural units in each CC, and the DS content of such word will be distributed in the cortical patch simulated by the EL. Significant evidence shows that the semantic content of words is distributed across cortical tissue (Huth et al., 2016). For example, a word like *top* can not only activate a region related with clothing and appearance, but it can also activate a region related with numbers and measurements and perhaps a region related with buildings and places. On the other hand, it has also been shown that words with similar semantic content are mapped in proximal regions of the cortex (Huth et al., 2012). For instance, in the right temporo-parietal junction a small patch of cortex responds to words like *wife, mother, pregnant, family, friends, brother* etc, while a very near patch responds to words like *family* and *wife* but also responds to—in certain way semantically related—words like *house, roommates, household*, and *owner*. There are compelling brain-inspired computational approaches which use SDRs to simulate how cortical brain activation represents the Distributional Semantic (DS) content of words (Webber, 2015).

In Figure 4, we illustrate how the word hours could activate a cluster of neural units which are representative of time phenomena such as pm, hour, and sometime, but it could also activate units which represent semantically related phenomena such as tense, which has to do with the form of a verb showing the time at which an action occurred, or the words arrive, waiting, and finished, which are also—more indirectly—related with time. Each CC in our approach has a representative model of the DS space and every column in the EL learns a complete model of such semantic representation. We rely on compelling brain-inspired computational theories in which every column in every region learns complete models of objects (Hawkins et al., 2019).

One important property to highlight in Figure 4 is that the advent of a word and its consequent semantic activation from word2vec will not determine a neural unit to fire, instead, it will bias the probability of such neural unit in doing so. That is, the more excited a neural unit is by its afferent dendrite, the more likely it is that such unit will become active. That is why neural units which are closer to the active word vector in the sub-space will tend to be more densely active than neural units farther apart, as Figure 4 shows. The stochastic behavior of cells in neural tissue is a widely used property not only in bio-inspired models (Harrison et al., 2005) but also in Artificial Neural Networks (ANNs) such as Deep Learning (DL) networks by means of techniques like dropout (Srivastava et al., 2014) which is used to reduce unwanted phenomena such as overfitting.

### 2.3. Lateral Sequential and Apical Coarse-Grained Syntactic Constraints

It is generally believed that—unlike cells in layer IV that respond to more shallow stimuli—cells in layer II/III are known to be *complex cells* that respond to sequence of motion or cells that respond to different translations of the same feature in an invariant way (Hirsch et al., 2002; George and Hawkins, 2009; Antolik and Bednar, 2011). For instance, cells in layer II/III in visual and barrel cortical areas—to a great extent—favor richer stimuli, such as motion in the preferred direction (Hirsch and Martinez, 2006). This is consistent with our proposal that layer II/III cells represent different patterns in the context of different sequences. They become active according to the context of the correct sequence.

**Figure 5:**
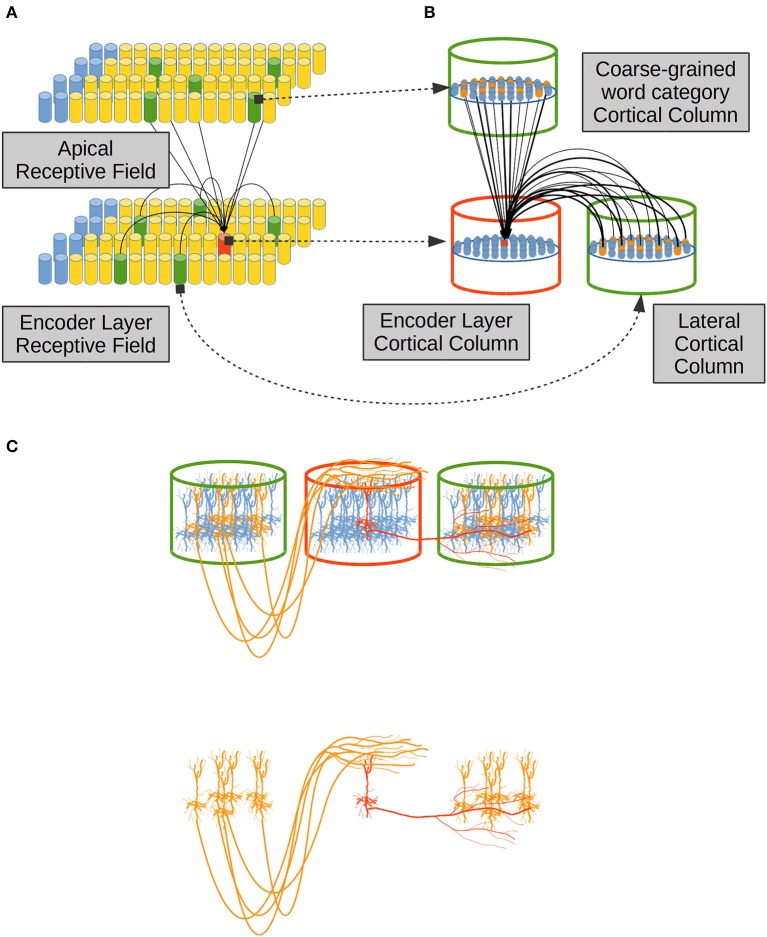
Distal dendrites in the EL. **(A)** Distal dendrites can be (i) lateral, linking neighboring CCs in the same cortical patch, and (ii) apical, which link CCs in different cortical patches. **(B)** A distal dendritic branch between the red CC and a green CC entails that every neural unit in the red CC is linked to a different subset of neural units in the green CC by means of potential connections. The subset of potential connections comes from a percentage of neural units inside the green CC. Such percentage is a tunable parameter for the CC. **(C)** Physical proximity of a dendritic branch from the red cell to axonal branches from yellow cells determines potential connections which could prosper becoming in established synapses depending on the activity among cells. Adapted from https://doi.org/10.1371/journal.pone.0217966 under CC-BY license.

Distal dendrites in each neural unit in the EL are classified in two sets: (i) lateral dendrites which connect a CC—in red in Figure 5A—to neighboring CCs in the same cortical patch—in green in the EL in Figure 5A. It is known that layer II/III pyramidal neurons axons travel several millimeters parallel to the layer II/III and/or layer IV boundary re-entering in layer II/III to make excitatory connections to pyramidal cells there (Lund et al., 2003; Bannister, 2005). Then (ii) apical dendrites which link a CC in the EL to CCs in another cortical patch. In the present work, apical dendrites bring information from a Receptive Field (RF) which establishes coarse-grained syntactic constraints in the system (Figure 5A).

A distal dendrite linking two CCs as in Figure 5A implies that each neural unit in a CC—in red in Figure 5B—is linked to a sub-set of units in neighboring CCs in the same cortical patch, or in other CCs in a foreign cortical patch, as shown in green in Figure 5B. Such sub-set of neural units is determined by the physical anatomical configurations of dendrites from the red neural unit in Figure 5B to the yellow neural units in green CCs, as shown in Figure 5C.

Figure 5C shows how lateral dendrites extend through cortex to link a neural unit to other neural units in neighboring CCs in the same cortical patch—the EL in our case. Apical dendrites on the other hand, receive information from CCs located in foreign cortical patches by means of extended axons which leave their cortical domain, traveling through white matter and finally entering the EL cortical patch up to the most superficial layer of cortex called L1, where apical dendrites from local cells extend.

**Figure 6:**
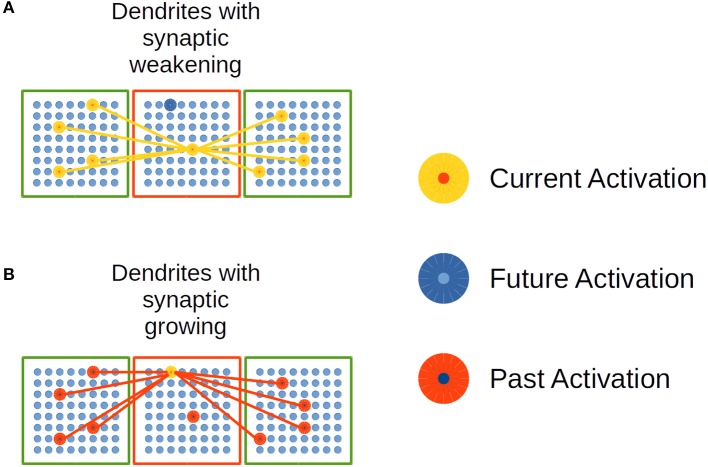
Synaptic growth in distal dendrites in the EL. **(A)** Dendrites with synaptic weakening. **(B)** Dendrites with synaptic growing. Adapted from https://doi.org/10.1371/journal.pone.0217966 under CC-BY license.

Figure 6 depicts the process of synaptic growth in distal dendrites. The red square represents a CC whose distal dendritic branches link its neural units to neural units in other CCs in green in the figure.

In Figure 6A, the current and simultaneous activation of neural units in linked CCs decreases the synaptic strength in the potential synapses in such dendrites. In Figure 6B, potential synapses among currently activated neural units and past activated ones are strengthened. This mechanism of synaptic growth simulates Spike-timing dependent plasticity (STDP) biological processes in cortex.

As already mentioned, lateral dendrites bring information from previous activity in neighboring CCs in the EL. This adds restrictions to the activation of neural units regarding previous activation in the same area. This would produce syntactic constraints which would bias the activation of certain patterns compared to others which are less coherent regarding the statistical sequential regularities immerse in the grammatical structure of the stimuli.

Apical dendrites bring information from foreign cortical patches. In order to provide such information, we generate three Sparse Distributed Representations (SDRs) which supply a coarse clue to the EL about three major word categories: (i) content words, (ii) function words and from content words we segregate, (iii) verbs. Previous research has shown that content-function word distinction is marked acoustically, phonologically, and distributionally in the speech infants hear in different languages (Shi, 1995; Shi et al., 1998). Even newborn infants can categorically discriminate word classes based solely on acoustic and phonological cues. Furthermore, such phonological cue can help older infants bootstrap into the acquisition of grammatical categories and syntactic structure (Shi et al., 1999). Lohmann (2017) empirically showed that noun-verb conversion can be determined via phonological properties of word class. In such regard, phonological cues can be employed for words of at least two syllables for the determination of the directionality of the noun-verb conversion. We consider that phonological constraints could provide a much richer and fine-grained correlated repertoire, yet we kept such constraints at a minimum in order to test the model's reaction using the minimal hint phonology could provide.

### 2.4. Cortical Activation in the Encoder Layer

**Figure 7:**
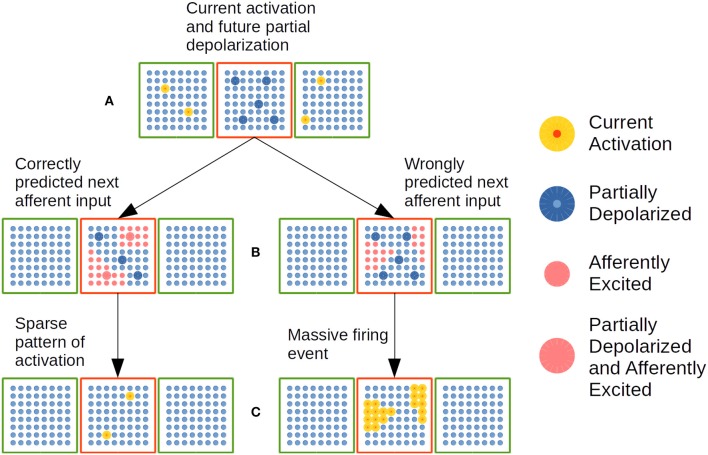
Dynamic cellular activation in a CC in the EL. A red cortical column is linked with two green cortical columns by means of distal dendrites. **(A)** Cellular activation in green CCs—highlighted yellow cells—puts neural units in red CC in a partially depolarized—predictive state highlighted in blue. **(B)** Cluster of neural cells activated by afferent inputs. Left: A substantial amount of partially depolarized cells are in the afferently excited cellular clusters. Right: There is no substantial amount of partially depolarized cells inside afferently excited cellular clusters. **(C)** CC with active cellular units highlighted in yellow. Left: Sparse pattern of cellular activation. Right: Massive pattern of activation. Adapted from https://doi.org/10.1371/journal.pone.0217966 under CC-BY license.

The dynamics of cellular activation in the EL is depicted in Figure 7. Repeated correlation among DS afferent, sequential lateral and coarse-grained word category apical constraints determines the strength of distal dendritic synapses in such a way that certain events will be more predictable than others. As long as the sequence of sentence constituents keeps a certain level of predictability with respect to the training material, activation patterns in the EL will return enough sparsity, and a phenomenon of repeated SDRs will be sustained throughout time. When unlikely events—with respect to training material—arise, sparsity cannot be sustained by the network and a phenomenon called Massive Firing Event (MFE) will emerge as a consequence of the inability of the network to correctly predict such events. As a result of the ignorance of the network to correctly predict an event, it activates all likely hypotheses given the semantic constraints. In this way, the EL loses the bias established by syntactic constraints, opening more hypotheses in order to receive subsequent lexical events in a better condition.

In Figure 7A, current activation of neural units in green CCs plus distal dendritic synapses established after learning, partially depolarize specific neural units in the red CC. Such partially depolarized units are set as predictable firing units for the arrival of the next lexical event. In Figure 7B, afferent semantic constraints from the next sentence constituent excite specific clusters of neural units in the red CC. On the left, such clusters of afferently excited units contain enough partially depolarized units which will activate before rival units in the excited clusters and—as a consequence of that—will be able to inhibit rival units activation as shown in Figure 7C on the left. In such way, the current lexical event is correctly predicted by the network. On the right side of Figure 7B, the afferently exited clusters do not contain enough partially depolarized units, which implies that the great majority of the units in the clusters are in very similar conditions to fire. This circumstance determines that all the units in the afferently excited clusters will fire producing a MFE as shown in Figure 7C on the right. Such event indicates that the network is not correctly predicting the sequence of lexical constituents in the sentence.

### 2.5. Experimental Setup

**Figure 8:**
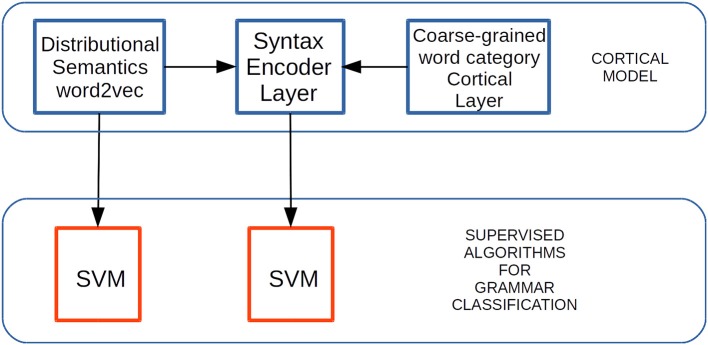
Experimental setup to test grammar classification task performance. Distributional Semantic (DS) constraints generated by means of word2vec are received by the EL afferent dendrites. Coarse-grained word category constraints are SDRs received by the EL apical dendrites. Grammatically-related word classification tasks are performed on both outputs—from word2vec and from the EL—by the SVM algorithm. Adapted from https://doi.org/10.1371/journal.pone.0217966 under CC-BY license.

The experimental setup used in this paper is depicted in Figure 8. Our main hypothesis is that cortical activation from the EL in response to sentence lexical constituents, will provide better information to the supervised algorithm to classify the grammatical function in such constituents than the information provided by word2vec. Therefore, we used the features delivered by word2vec and by the EL in response to each sentence constituent in order to train both classifiers shown in Figure 8. Then, we tested the trained algorithms using different corpora to the one used for training.

We used supervision by means of the SVM classification method, receiving the outputs from each algorithm. We did this to test the generalization properties in the grammatical features abstracted by the EL in comparison with the grammatical features returned by word2vec (Figure 8). In the present work, we used a package called Library for Support Vector Machine (LIBSVM) (Chang and Lin, 2011; lib, 2016). We trained and tested the SVM classifiers using five-fold cross-validation, and configured them to use a linear kernel with one parameter *C*, which we swept to find the best trained model for each classifier.

We implemented an EL with 225 CCs arranged in a two-dimensional array of 15 × 15 CCs. Each CC was automatically distributed using individual locations along its afferent, lateral, and apical inputs in a uniform way. Each CC received afferent information by means of two-dimensional afferent receptive fields of 9 × 29 components centered at individual locations over a 10 × 30 word2vec space. We enabled the wraparound property in order to make each receptive field span the entire word2vec array. We also instructed each column to receive only 31 inputs, which is a minor percentage of such receptive field. Individual afferent inputs for each CC were chosen randomly during the EL initialization process.

For this model instance we used distal lateral and apical dendritic branches. We configured each CC to have a lateral receptive field with 9 × 9 neighboring CCs and to receive information from 72 of the 81 CCs in the receptive field—a 90% of the receptive field. In reference to apical dendrites, we configured each CC to have an apical receptive field of 11 × 11 foreign CCs and to receive information from 108 of the 121 CCs in the receptive field—also a 90% of the receptive field.

Each CC was composed of a two-dimensional array with 15 × 15 (225) neural units and each unit in a column could be potentially connected with only six neural units from each linked neighboring column. Each neural unit in a CC ended up with 72 lateral dendritic branches with six potential connections each (432 distal lateral potential synapses per cellular unit) and with 108 apical dendritic branches with six potential connections each (648 distal apical potential synapses per cellular unit). That is, each neural unit in a CC ended up having 1,080 distal potential synapses. Such potential synapses were randomly chosen for each neural cell and for each dendritic branch in the cell during the Encoder initialization procedure. The EL consisted of 50,625 cellular units with 1,569,375 proximal synapses and 54,675,000 distal synapses. It is important to highlight that distal synapses represented potential connections from which only a small percentage had a significant synaptic weight as to be considered as an established connection. Weak synapses were periodically pruned by means of homeostatic processes in the network, leaving distal dendrites with a sparse connectivity in the receptive fields. The sparseness in such connectivity matrices could exceed 90%.

The fictitious Cortical Layer (CL) from which the EL received apical constraints—in the form of coarse-grained word category SDRs—had the same columnar and cellular configuration than the EL.

The training procedure consisted of two stages and for each stage the EL received the same corpus twice. During each learning stage, certain parameters—such as the learning rates in proximal and distal synapses and the lateral intra-column interaction—were exponentially and progressively decreased from an initial value. The same parameters were also decreased for each successive stage. An additional stage was executed with the learning parameters fixed.

The sparsity in the activation for each CC—even CCs in the coarse-grained word category Cortical Layer (CL)—was 99% (just 2 neural units out of 225 could be active for normal activation events). On the other hand, the afferent excitation affected 10% of the units inside the clusters in each CC (22 neural units, which could be activated in case of a MFE; Figure 7).

In order to train the model we used a corpus from the WikiSplit dataset by Google (Botha et al., 2018). This dataset was constructed automatically from the publicly available Wikipedia revision history. The complete dataset contains one million English sentences, each split into two sentences that together preserve the original meaning, extracted from Wikipedia edits. In order to train the model we used a corpus called test from the dataset. The corpus has 14,980 sentences. We cleaned the corpus erasing punctuation marks to get a file in which each sentence is a sequence of individual words in line.

We tagged each word in the corpus with its grammatical function in the sentence context. To that end we used Enju natural language parser for English [Enju 2.4.4 Copyright (c) 2005-2010, Tsujii Laboratory, The University of Tokyo]. This parser has a wide-coverage probabilistic Head-Driven Phrase Structure Grammar (HPSG) (Miyao, 2002; Yusuke and Jun'ichi, 2002; Miyao and Tsujii, 2005, 2008; Miyao et al., 2005; Ninomiya et al., 2006a, 2007), as well as an efficient parsing algorithm (Tsuruoka et al., 2004; Ninomiya et al., 2005, 2006b; Matsuzaki et al., 2007).

Enju can effectively analyze syntactic/semantic structures of English sentences and provide the user with phrase structures and predicate-argument structures. Those outputs would be especially useful for high-level NLP applications, including information extraction, automatic summarization, question answering, and machine translation, where the “meaning” of a sentence plays a central role (Enju, 2019).

We analyzed the complete corpus grammatically by means of Enju in its stand-off format. In this format, the span of each tag is represented with the position in the original input sentence, each line representing a tag. The label of a tag (e.g., “cons” and “tok”) is output first, and the rest represents the attributes. A constituent is tagged by cons while each word is tagged by tok. The attribute “cat” represents the phrase symbol of the constituent. The attribute “pos” represents a part-of-speech and—inside tok—the attribute cat represents the same information as in cons.

To tag the grammatical function of the words in each sentence we used part of the information returned by Enju. We specifically used tok tags from which we extracted the attributes cat and pos. The conjunction of those two attributes formed the grammatical function with which we tagged each word in the sentence context for all the corpus. In this way, we ended up having 113 different grammatical categories.

Once we had all the words in the corpus tagged, we synchronized apical SDRs in the training stage in such a way that nouns, adjectives and adverbs were correlated with the *content word* SDR. Articles, auxiliaries, demonstratives, quantifiers, prepositions, pronouns, conjunctions, etc, were correlated with the *function word* SDR, and the rest of the tagged words were correlated with the *verb* SDR.

First, we trained the model, and then we ran it in inference mode. In such mode, the EL processed the information with its learning properties disabled. In this manner, during inference, the EL did not modify its synapses and just returned patterns of activation in response to the stimuli it received. We then used the outputs from word2vec and from the ELs in inference mode to train the SVM classifiers using the grammatical tags we obtained from Enju.

## 3. Results

We used the outputs from word2vec and from the EL in inference mode to train the SVM classifiers shown in Figure 8. We used the outputs from such algorithms in response to 150, 300, and 600 sentences from the corpus. The cross validation training performances are shown in Table 1.

**Table 1:** SVM cross validation training results.

	**Word2vec (%)**	**Encoder layer (%)**
150 sentences	84.03	89.40
300 sentences	83.35	90.47
600 sentences	80.96	90.79

We then tested the classification accuracy of each SVM algorithm—the one trained using 150 sentences, the one trained using 300 sentences, and the one trained using 600 sentences—using the outputs from word2vec and the EL in response to different sentences—not used to train the classifiers. We did so for 10 different sets of sentences in each case—i.e., 10 different corpora with 150 sentences, 10 different corpora with 300 sentences and 10 different corpora with 600 sentences.

**Figure 9:**
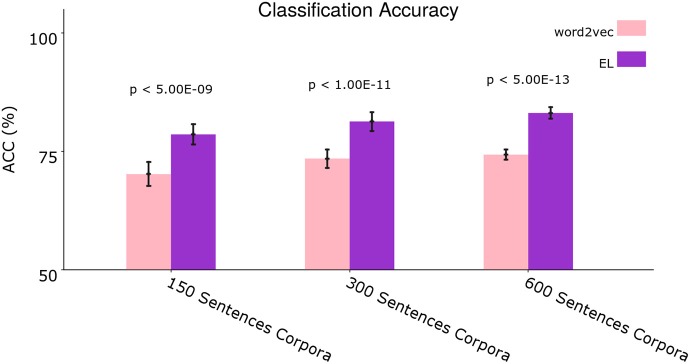
Average classification accuracy. Average classification accuracy of grammatically grounded features returned by word2vec vs. features returned by the EL for three experimental conditions. The *p*-values correspond to two-tailed paired *t*-tests (Holm–Bonferroni validated); each for 10 different corpora. Error bars depict 95% Confidence Interval values. Adapted from https://doi.org/10.1371/journal.pone.0217966 under CC-BY license.

Figure 9 shows the average classification accuracy returned by the tests in each case—i.e., 150, 300, and 600 sentences corpora.

We performed two-tailed paired *t*-tests for 10 different corpora from the dataset. Given that we conducted three *t*-tests for the grammar classification task (i.e., 150, 300, and 600 sentences), we performed Holm–Bonferroni corrections with a correction factor of three in order to reduce the probability of type I and type II errors in the tests (Hommel, 1988). As can be seen in Figure 9 the EL performed significantly better than word2vec in all the experimental conditions.

### 3.1. Individual Sentence Analyses

**Figure 10:**
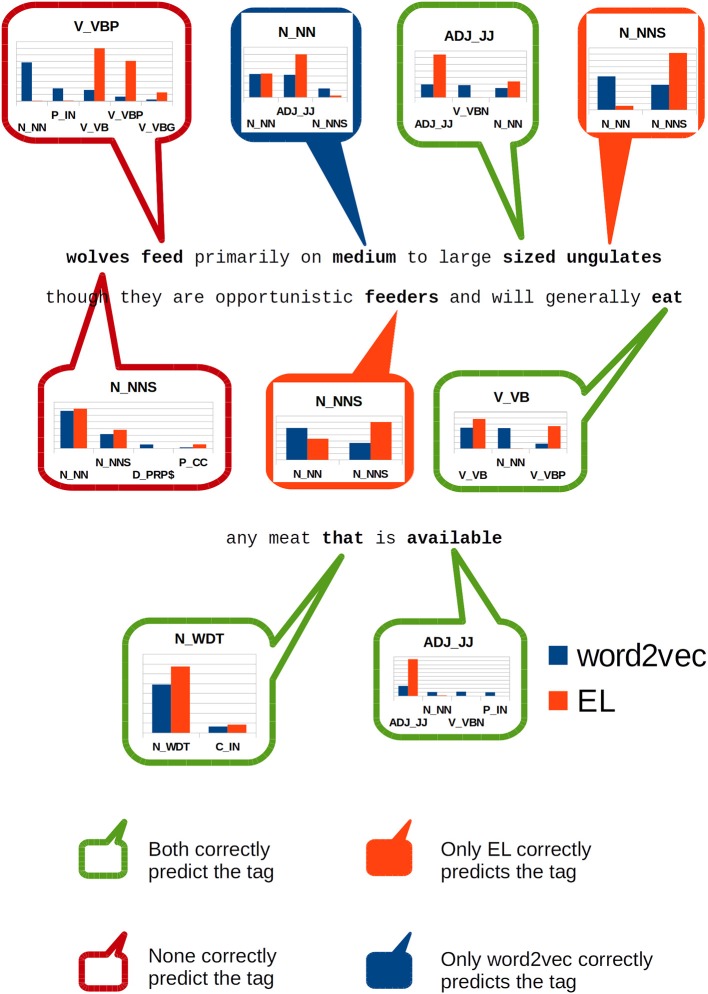
Classification of individual constituents within the sentence: wolves feed primarily on medium to large sized ungulates though they are opportunistic feeders and will generally eat any meat that is available.

With the aim of illustrating the results showed by Figure 9, Figure 10 shows how word2vec and the EL serve SVM algorithms for grammar classification tasks within the context of the sentence:

wolves feed primarily on medium to large sized ungulates though they are opportunistic feeders and will generally eat any meat that is available.

In Figure 10, we highlight specific words in the sentence and show how SVM models classify them using the information produced by each algorithm.

The first word (wolves) is incorrectly classified by both algorithms losing the number sense in the lexical entry which is correctly detected by our gold standard—Enju. The second word (feed) is a verb, non-3rd person singular present according to our gold standard. Both algorithms incorrectly classified such constituent too, but this time there was a clear difference regarding the information each algorithm provided the classifiers. On the one hand, word2vec provided features to which the classifier assigned a maximum of 29% likelihood of being a singular noun. On the other hand, the EL provided features to which the classifier assigned 39% chance of being a verb in base form, but it also assigned 30% likelihood to the same tag assigned by the gold standard—which is the correct one for this case.

The word medium which clearly acts as an adjective in this sentence, is missclassified by our gold standard as a singular noun; hypothesis to which word2vec endorses. Nonetheless, the EL provides information to the SVM such that it assigns almost 60% probability that this sentence constituent is an adjective.

The word sized acts as an adjective in this sentence too. This constituent is correctly classified by both algorithms, but the EL provides activation which gives the SVM algorithm more than 64% confidence about its classification, while word2vec provides features that turns the SVM classification considerably more undetermined: it assigned 19% to the correct tag, but in addition it also assigned 18 and 13% to the tags “verb in past participle” and “singular noun,” respectively.

In the words ungulates and feeders the EL provided enough information to the SVM algorithm as to make it assign a high probability—more than 90% for ungulates and more than 60% to feeders—to the correct tags in both cases. On the other hand, word2vec lost the number sense attributing a singular noun tag to both constituents.

The word eat is correctly classified by both algorithms but the EL assigns the highest probability to the correct tag—more than 48%—while word2vec spreads chance out along a larger range of tags including nouns.

Finally, the word available was correctly classified by both algorithms, but the EL assigned by far the highest probability to the correct tag—more than 93%—while virtually neglecting alternative tags as viable options. On the other hand, word2vec assigned a scarce 25% chance to such tag, distributing the probability throughout a much larger range of tags.

### 3.2. Testing an EL With Stripped Lateral Connections

In order to analyze the contributions provided by the two different distal dendritic trees in the model in regards to the classification task, we tested an instance of the EL but this time we stripped all its lateral connections—we call this EL instance as Encoder Layer with Stripped Lateral Connections (EL SLC). We trained and tested this instance using the same procedure depicted above. The cross validation training performances comparing the normal version of the EL with the new instance without lateral connections are shown in Table 2.

**Table 2:** SVM cross validation training results on the comparison between an EL SLC and a normal EL.

	**EL SLC (%)**	**EL (%)**
150 sentences	88.03	89.40
300 sentences	89.16	90.47
600 sentences	87.07	90.79

As before, we tested the classification accuracy of each SVM algorithm–the one trained using 150 sentences, the one trained using 300 sentences and the one trained using 600 sentences—using the outputs from the EL SLC and the EL in response to different sentences—not used to train the classifiers.

**Figure 12** shows the average classification accuracy comparing the normal EL with the EL SLC.

**Figure 11:**
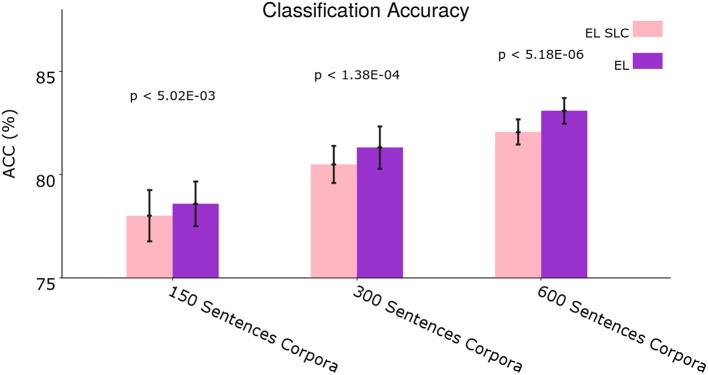
Average classification accuracy. Average classification accuracy of grammatically grounded features returned by the EL SLC vs. features returned by the EL for three experimental conditions. The *p*-values correspond to two-tailed paired *t*-tests (Holm–Bonferroni validated); each for 10 different corpora. Error bars depict 95% Confidence Interval values. Adapted from https://doi.org/10.1371/journal.pone.0217966 under CC-BY license.

Once again we performed two-tailed paired *t*-tests for 10 different corpora from the dataset and applied Holm–Bonferroni corrections with a factor of 3. As Figure 11 shows, the normal EL performs slightly better than the EL SLC. This global difference in performance is statistically significant for all the experimental conditions according to the *t*-tests. A disaggregated statistical analysis showing how distal lateral dendrites contributes to the classification of different syntactic categories is developed in section 3.3.

### 3.3. Segregated Statistical Analyses on Individual Grammatical Categories

We trained two SVMs—one receiving word2vec outputs and the other receiving the EL outputs on a different corpus to the corpora used in the previous experiments. SVM cross validation training results on the 600-sentences-corpus were 83.04% on word2vec and 91.74% on the EL.

We performed a segregated statistical analysis on each grammatical category produced by Enju. We compared the performance on word2vec vs. the EL for each category. To that end we averaged the performance on each tag from 10 different corpora—each of 600 sentences—which were composed by different sentences to the ones used to train the classifiers. For each tag we computed *p*-values corresponding to two-tailed paired *t*-tests; each for the 10 different corpora.

**Figure 12:**
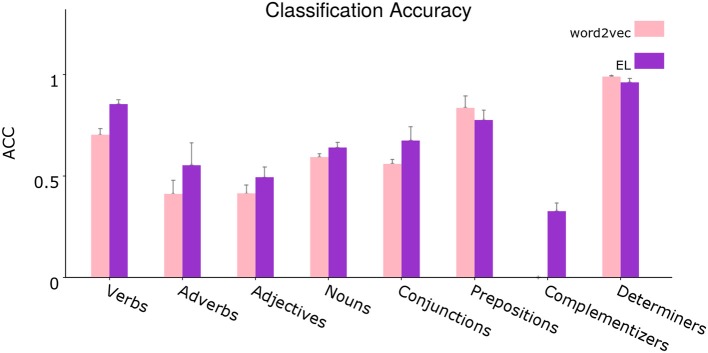
Segregated average classification accuracy. Average classification accuracy of grammatically grounded features returned by word2vec vs. features returned by the EL for a *coarse-grained* clustered version of the grammatical tags. Error bars depict 95% Confidence Interval values. Adapted from https://doi.org/10.1371/journal.pone.0217966 under CC-BY license.

Figure 12 shows the results for the tests comparing word2vec and the EL. In the figure we show a clustered version of the grammatical categories produced by Enju. Since the frequency of occurrence of some categories was of negligible value, they were not included in the analysis.

The figure shows a disaggregated version of the evaluation shown in Figure 9. From the statistical analysis, the EL bootstraps over word2vec significantly for the verbal tags V_VBP, V_VBN, V_VBD, V_VBZ, V_VBG, and V_VB, for the adverbial tags ADV_RB, ADV_IN, and ADV_RBR, for the noun tags N_NN, N_NNS, N_FW, N_PRP, N_DT, and N_CD, for the adjectives ADJ_CD and ADJ_JJ, for the complementizers C_TO and C_IN, for the coordination conjunction CONJ_IN and for the prepositional tag P_IN. On the other hand the EL gets statistically significant reduced performance respecting word2vec for the determiner tag D_PRP$, for the subordination conjunction SC_IN, for the adjective ADJ_JJS, and for the prepositional tag P_TO.

It is important to highlight some specific cases in which the EL gets significant classification performance without word2vec performance from which to bootstrap. That is, in such cases word2vec had a performance of 0%. The grammatical categories for which this situation is given are for instance the complementizers C_TO and C_IN, the adverbial tags ADV_IN and ADV_RBR, the coordination conjunction CONJ_IN, and finally the nouns N_DT and N_CD.

**Figure 13:**
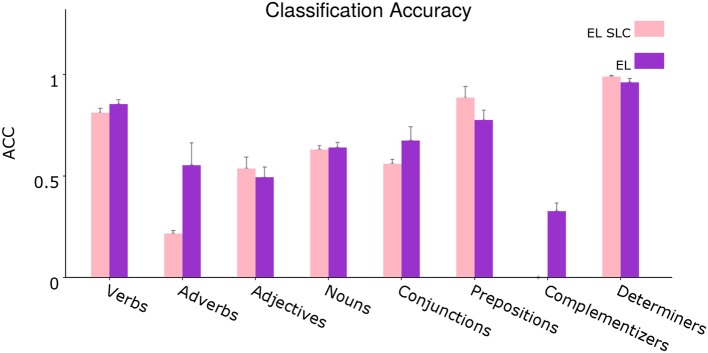
Segregated average classification accuracy. Average classification accuracy of grammatically grounded features returned by the EL SLC vs. features returned by the EL for a *coarse-grained* clustered version of the grammatical tags. Error bars depict 95% Confidence Interval values. Adapted from https://doi.org/10.1371/journal.pone.0217966 under CC-BY license.

In Figure 13, we conduct the same procedure developed before, but this time corresponding to a disaggregated version of the evaluation shown in Figure 11. In this analysis we evaluate how distal lateral dendrites contribute to the classification performance of individual syntactic categories.

From the statistical analysis, distal lateral dendrites produce a significant improvement in performance for the verbal tags V_VBP, V_VBD, and V_VB, for the adverbial tags ADV_RBS, ADV_IN, ADV_RB, and ADV_RBR, for the complementizers C_IN and C_TO, for the noun tags N_PRP, N_DT, and N_CD and for the coordination conjunction CONJ_IN. On the other hand, distal lateral dendrites reduce the classification performance for the determiner D_PRP$, for the noun N_NN, for the adjective ADJ_JJR, for the verb V_VBZ, for the subordination conjunction SC_IN, for the particle PRT_RP and for the prepositional tag P_TO.

## 4. Discussion

In this paper we introduced a computational model inspired in specific features of brain cortical tissue whose outcome mimics linguistic relevant selection mechanisms found in brain cortex (Rego and Bryant, 1993; Gibson and Pearlmutter, 1998; Hagoort, 2005; Lopopolo et al., 2017).

By means of the experimental results presented here we show how the convergence of linguistic constraints from different sources, improves the classification of grammatical functions carried by constituents within a sentence context.

For instance, when analyzing a specific sentence, both algorithms lose number sense in the first word (wolves). Except for such case, the EL catches the number sense in the rest of the cases in which plural nouns appear (ungulates and feeders), while word2vec continues losing number sense in those examples too. As can be seen in Figure 12 and from the statistical analysis conducted in section 3.3, the EL significantly outperforms word2vec in the classification of singular and plural nouns.

In our implementation, coarse-grained word category constraints from apical dendrites do not provide enough information to the EL to make such distinction. Initially our group contemplated the possibility that syntactic constraints coming from lateral dendritic activation within the sequence of constituents, could fulfill the information needed by the EL to constraint the choices among different unification options. In such way, the EL, engaging such constraints, could have ended up activating only the most suitable option given the information received. In this example, Distributional Semantic (DS) information from word2vec does not suffice to distinguish the number attribute in nouns, but the incorporation of syntactic constraints such as the adjectives preceding nouns could have biased the probability toward the plural attribute. Furthermore, larger distance dependencies such as the word are in the phrase are opportunistic feeders, could have influenced the classification given the sequential properties incorporated in the EL (Cui et al., 2016). Since syntactic constraints do not appear in the first word of the sentence, the EL could have made the same kind of mistake than word2vec, and neither DSs nor coarse-grained word category constraints would provided sufficient information as to catch the plural attribute in the noun.

In order to test such hypothesis, in section 3.2 our group tested a version of the EL without lateral dendritic arms. Figure 11 shows that lateral dendrites in our model play certain role in the integration of sequential information given the statistical significance returned by the experiments. Yet, a disaggregated analysis of Figure 11—conducted in section 3.3—allowed us to conclude that the improvement in the distinction between singular and plural nouns comes from the combination of afferent and apical constraints, and not from the sequential information provided by lateral constraints. This phenomenon can be seen in section 3.3, in which the performance in the classification of singular nouns is reduced with the incorporation of lateral dendritic trees while the performance on the classification of plural nouns is not significantly affected.

The classification of verbs in the sentence (feed and eat), clearly shows the importance of the coarse-grained word category constraints from apical dendrites (Shi, 1995; Shi et al., 1998, 1999, 2006; Lohmann, 2017). In such regard, word2vec confers a very high weight to nouns in both examples, completely misclassifying the word feed as a singular noun and giving almost the same probability (33.9 vs. 33.3%) to the tags verb in base form and singular noun when the verb eat appears. The EL on the other hand, virtually disregards the classification of such verbs as nouns, misclassifying feed as a verb in base form although providing a high chance to the correct tag (the second highest). The EL also produces a correct classification of eat, providing a high probability to the correct tag.

In section 3.3, Figure 12 shows that grammatical constructions for verbs are classified significantly better by the EL. The unique exception is for the case of modal verbs (V_MD) as can be inferred from the statistical analysis. From Figure 13, we can see that lateral dendrites contribute significantly to the classification of verbs. From the statistical analysis specific improvements are for the *verb, non-3rd person singular present* (V_VBP), *verb, past tense* (V_VBD), and *verb, base form* (V_VB).

Adjectives are quite remarkable as can be seen in Figure 10. Even though the adjectives sized and available are correctly classified by both algorithms, there is a notorious difference in such classifications which can be appreciated in the soundness with which the EL attributes chances to the correct tag in both cases. On the other hand, word2vec is indeterminate, attributing a very low chance to the correct tag, especially in the case of sized, in which the algorithm attributes almost the same probability to the tags adjective, verb in past participle and singular noun. Another important example is given with the word medium. Even when the appearance of such word counts as a success for word2vec, the reality is that the EL produces a correct classification of it as an adjective, and such classification is sound due to the high probability assigned to the tag. From Figure 12, we can see that the EL improves the classification of adjectives significantly. Figure 13 shows that this improvement does not come from the contribution provided by distal lateral dendrites.

It is important to highlight some specific cases for which the EL builds its own classification performance from a word2vec performance of 0%. The most prominent cases are for the following tags: C_TO, with typical examples like …*the voice* ***to****renew…* or …*released* ***to****radio…* and CONJ_IN, with typical examples like …*as well* ***as***… or …*rather* ***than***…. Moreover, the classification performance in such cases is completely sustained by distal lateral dendrites as can be inferred from the statistical analysis conducted in section 3.3.

For some adverbs, such as *superlative* (ADV_RBS) with examples like *the third* ***most*** *common…* or *his* ***most****serious poem…, subordinating conjunction* (ADV_IN) with examples like *after wandering* ***around****he discover…* or …*soon* ***after***and *adverb, comparative* (ADV_RBR) with examples like …*to* ***better****measure future cash flow* or …*her autobiography* ***more****important than her poetry*, the EL classification performance is fully sustained by distal lateral dendrites.

Adverbs (ADV_RB) with examples like …*that is* ***almost****completely black* or *it was released* ***only****in australia*; are classified significantly better by the EL and distal lateral dendrites—even when not exclusively—contribute significantly to such classification performance.

Compared to word2vec, the EL activates fewer phrasal configurations, narrowing down the spectrum of alternative binding candidates. It assigns higher probability values to fewer tags and generally such tags are the correct ones or closer to the correct ones. This fact is supported by the higher hit rate of the EL compared to word2vec (Figure 9).

Regarding related works in the field, Pulvermüller and Knoblauch (2009) refuted the *rule-free* claim attributed to neural networks, by demonstrating the emergence of discrete neuronal aggregates in a brain-inspired model previously developed by the same authors (Knoblauch and Pulvermüller, 2005). The model includes a network of several *sequence detectors* and demonstrates that associative Hebb-like synaptic plasticity rules can learn word sequences and form neural representations of grammatical categories. Although such a model can function as a basis of syntactic rule application and generalization, it was not tested on more complex sequences of syntactic constructions inside complete sentences as the ones used in this research.

Wennekers et al. (2006) introduced a modeling approach in which multiple area networks are built of anatomically identical features. Similarly to the model we present in this paper, the architecture of such network introduces cells which include feedforward, feedback and lateral connections which are set up by means of associative principles motivated by Hebb's rules (Hebb, 1950). In a more recent work, using the same modeling principles Tomasello et al. (2017) developed a physiologically plausible artificial neural-network that replicated sensorimotor, multimodal, and language areas. The experiments reported emergent *semantic circuits* for object- and action-related words which exhibited category-specificity in modality-preferential areas. Even though results from both works can be compared with real experimental data—thanks to the realistic neurocomputational facet of the models—the experimental profiles were mainly centered on statistical correlation measurements, over semantic and more limited aspects of grammar—such as action- and content-words. The learning of more complex syntactic structures was not in the scope of such research. In general, Wennekers et al. (2006) manifested the acquisition of syntactic structures as a difficult problem in assembly networks. Furthermore, no ML like recognition tasks were conducted, the corpora used for the experiments were artificially generated and the models were not tested on classification invariance. Having said that, it is important to highlight that the use of DSs—such as word2vec—as input to our model limits its biological plausibility in comparison with the research conducted by Wennekers et al. (2006). Future work in our research will be directed toward improving this aspect.

A computational model that assigns thematic roles to sentence constituents has been previously developed by John and McClelland (1990). The model disambiguates words, instantiates vague words, and elaborates implied roles. Recently, this computational approach was used to explain the N400 event-related brain potential presenting a computationally explicit account for the emerging representation of sentence meaning (Rabovsky et al., 2018). The model succeeded in capturing diverse empirical neural responses, showing that essential aspects of human language processing can be effectively represented by a proper connectionist approach. Nevertheless, the model lacked a mapping of neurophysiological characteristics in cortical dynamics and used optimization algorithms (i.e., backpropagation) which are difficult to map in neural tissue. While we recognize some recent progress in using backpropagation as a plausible attribute of neural tissue–for instance Lillicrap et al. (2016) showed that even when the feedback and feedforward weights do not match, backpropagation still works–our work suggests that it may not be necessary to introduce the extra computational and algorithmic complexity needed by such mechanisms. Furthermore, in some cases (Bengio et al., 2017), several physiological requirements—not proven yet; such as the fact that the average soma voltage potential is insensitive to changes in the input firing rates when the neuron is saturated—have to be fulfilled before considering such research as truly plausible.

Dominey et al. (2009) on the other hand, incorporated the functional neurophysiology of sentence comprehension (along with non-linguistic sequence processing), in a neural network model whose architecture was constrained by Cortico-Striato-Thalamo-Cortical (CSTC) neuroanatomical connectivity and functional imaging data. The model was able to learn and perform several types of language and artificial syntax tasks. Their approach includes the interaction among several BA involved in language processing–such as Brodmann Area (BA) 47, 45, and 44/6 in the LIFG. Nonetheless, such model is also forced to choose the correct options through supervised error-driven learning methods. Such methodology, assumes the existence of internal *teaching signals* in the brain. Teaching signals are needed to force the output layer to the correct answer, enabling the network to backpropagate the errors.

Michalon and Baggio (2019) developed an explicit algorithmic implementation of a parallel processing architecture that explains how syntactic and semantic information processing can interact selectively during language comprehension. The architecture advances toward the organization of language in the brain focusing in the articulation between syntax and semantics and the essence of prediction in language comprehension. The work is clearly inspired by the psychology and neuroscience of language, but it does not incorporate biologically inspired features of cortical neural computation in its implementation.

In the present work the computational model developed, is inspired in the biology of the mammalian neocortex and simulates cortical tissue incorporating columnar organization, spontaneous micro-columnar formation, SDRs, and adaptation to contextual activation. In addition, different roles to proximal and distal dendritic configurations simulating pyramidal cells are assigned. We incorporate important physiological and anatomical phenomena, such as the consideration of dendritic branches as active and independent processing elements, the stochastic activation of brain cells and MFEs originated by prediction failures in the network manifesting as the activation of many neurons in a CC impairing SDRs formation—among others. Most ANNs, such as those used in previous works (John and McClelland, 1990; Dominey et al., 2009; Rabovsky et al., 2018; Michalon and Baggio, 2019), use artificial neurons without considering active dendrites and with an unrealistic low number of synapses, thus missing fundamental functional properties present in the brain. Furthermore, unlike established computational models, the model presented here does not incorporate optimization methods such as those found in supervised or reinforced algorithms. Even though influential research lines underpin the idea of *credit assignment* supporting backpropagation processes in cortex (Guerguiev et al., 2017), so far there is not enough evidence to justify the inclusion of such complex process in the brain. Moreover, our concerns regarding backpropagation in brain tissue go beyond the complexity of its algorithmic implementation. These implementations require the existence of teaching signals. Although there is evidence that animals can represent desired behavioral outputs with internal goal representations (Gadagkar et al., 2016), it is unknown whether teaching signals indeed exist in the brain. On the other hand, although very new and compelling algorithmic approaches such as BERT (Devlin et al., 2018) and GPT-2 (Vaswani et al., 2017; Radford et al., 2019) are making far-reaching changes in NLP in general, they continue needing complex optimization algorithms of difficult justification in brain cortex. Furthermore, they apply *Attention* mechanism without restriction demanding the full availability of all words in input sentences without providing clear explanations of memory mechanisms to sustain such phenomena in brain.

In the present work, we replace *teaching signals* used in other systems–specially needed by backpropagation-like optimization—by the uniform and simple correlation of SDRs activation coming from different cortical patches. Our hypothesis is that when noise is impairing the smooth individualization of a pattern coming from one source, the brain correlates such information with information coming from other sources in which the noise has not been too detrimental for the pattern that the subject seeks to classify. In the present model, when Distributional Semantic (DS) information from afferent dendrites is not sufficient for grammatical disambiguation of a sentence constituent, coarse-grained word category information from apical dendrites helps in such disambiguation. In the case that coarse-grained word category clues cannot compensate the lack of DS information, syntactical constraints from lateral dendrites finally come in handy. Functional connectivity across different cortical areas has been shown to facilitate speech comprehension when the intelligibility of the speech signal is reduced (Obleser et al., 2007).

On the other hand, the lack of reciprocal and/or recurrent anatomical connectivity among some cortical areas in our simulations is merely a feature of the model in its current state of development. Since DSs are provided by word2vec we are neither able to inject backward signals from the EL nor able to implement recurrent connectivity inside such standalone model. In the case of coarse-grained word categories, their static SDRs are neither able to be enriched by recurrent connectivity nor by backward connections from the EL module. We are aware that reciprocal connectivity between different areas as well as recurrent connectivity inside the same area is a repeated pattern in cerebral cortex. Therefore, future and more complex implementations of the model will include our own Distributional Semantic (DS) preprocessor—instead of word2vec—and coarse syntax generated from phonetic cues by means of the utilization of the computational hypotheses settled in Dematties et al. (2019). Such model will be enriched by reciprocal and recurrent connectivity in all their areas.

In this research we use some features of the information processing gradient discovered in the LIFG as a guidance to explain a plausible interaction of different lexical constraints in cortex. In this complex region of the neocortex information coming from BAs 47 and 45 is involved in semantic processing (Zhang et al., 2004; Carli et al., 2007; Newman et al., 2010; Goucha and Friederici, 2015) and we use it as the DS input gateway to the EL. It is important to highlight though that there are alternative pathways—beyond BA 47—in which semantic information is processed such as the temporal and inferior parietal lobes—among others (Binder and Desai, 2011).

In fact, many other sources of information—which turn out to be useful for early infant language acquisition—have not been yet considered in our computational approach. For instance, it has been shown that iconic gestures boost speech comprehension under adverse listening conditions (Holle et al., 2010). Neurocognitive studies of motor representations of speech sounds, action-related language, sign language, and co-speech gestures are also tightly coupled to the language system (Willems and Hagoort, 2007). Future work in the model will be directed toward integrating information from more sources than the ones presently used. We will also enrich coarse-grained word category information from apical dendrites with direct phonological cues to procure a more holistic linguistic integration. With this implementation we will also be able to apply backward connectivity possibly derived from BAs 45/44 to BA 6 and also to include recurrence in specific stages of the model related to coarse-grained syntax and phonology. For future developments of this work, we will add reinforced mechanisms to the model, including neuromodulator-like effects in the algorithm which could significantly enhance performance.

Finally, it is important to note that some of the assumptions claimed in this paper are computational hypotheses to be assessed by future research in the field. For instance, the fact that our modeling approach is inspired in differences between synapses across neural dendrites in cortex, does not imply that the function these synapses have in our model is the same in real neurons, neither that it is a necessary ingredient of our model. Specially designed experimentation has to be conducted in the future in order to elucidate such aspects. In the same line, the assignment of coarse-grained syntax information to apical dendrites does not suggest a structure-function relationship between aspects of syntax and apical dendrites in neurons. We are gathering biologically relevant claims and accounting for them in our model implementation. They are only comparable features which this work suggests of interest for future research and ML inspiration.

## 5. Conclusion

This research brings a novel modeling approach on how cortical activation for linguistic constraints could produce grammatical discrimination emergence in sentence constituents. The flow of information of a linguistic processing gradient is mapped in the cortical dynamics of a computational approach which simulates particular characteristics evaluated as suitable for linguistic computations in human neocortex. We introduce a biologically inspired computational model which incorporates specific features from the mammalian cortex. Our model utilizes Hebbian-like rules—assisted only by the gradual decrease of certain learning parameters. Yet, the leaning mechanisms in the model do not involve optimization mechanisms extensively used in prevalent Machine Learning (ML) algorithms, but of inconclusive evidence to support its justification in cortical tissue. We use such model inspired by unification operations at semantic and syntactic levels of language on the cortical Left Inferior Frontal Gyrus (LIFG). We show how cortical Sparse Distributed Representation (SDR) activation features returned by our model are well suited to attain classification of lexical grammatical functions of words bootstrapping Distributional Semantic (DS) features returned by word2vec. We evaluate this research as valuable for future and more brain-inspired ML applications of NLP as well as a complementary validation for psycho-linguistic theories of language processing.

## Data Availability Statement

All the data in this work are available from Zendo (https://zenodo.org/record/3653180, https://zenodo.org/record/3374889).

Regarding datasets:

The file Corpora.txt keeps the corpus used to train the model and the different instances of the classifier. It is basically a text file with one sentence per line from the original corpus called test.tsv available at https://github.com/google-research-datasets/wiki-split.git. We eliminated punctuation marks and special characters from the original file putting each sentence per line.

Enju_Output.txt holds the outputs generated by Enju in -so mode (Output in stand-off format) using Corpora.txt as input. This file has basically a natural language English per-sentence parse with a wide-coverage probabilistic for HPSG grammar.

The file Supervision.txt keeps the grammatical tags of the corpus. This file holds a tag per word and each tag is situated in a single line. Sentences are separated by one empty line while tags from words in the same sentence are located in adjacent lines.

The file Word_Category.txt carries the coarse-grained word category information needed by the model and introduced in it by apical dendrites. Each word in the corpus has a word-category tag which provides additional constraints to those provided by lateral dendrites. This file contains a tag per word and each tag is situated in a single line. Sentences are separated by one empty line while tags from words in the same sentence are located in adjacent lines.

The file SynSemTests.xlsx keeps all the grammar classification results as well as the statistical analysis in the classification tests.

The file ModelsComparison.xlsx keeps all the grammar classification results as well as the statistical analysis in the classification tests for the comparison of a normal instance of the Encoder Layer vs. an instance of the Encoder Layer with stripped distal lateral dendrites.

The file IndividualTaggingPerformance.xlsx keeps all the disaggregated grammar classification results as well as the statistical analysis in the classification tests for word2vec vs. the Encoder Layer and for a normal instance of the Encoder Layer vs. an instance of the Encoder Layer with stripped distal lateral dendrites.

The file Frontiers_Supplementary_Material.pdf provides details about the algorithmic formulation of the basic computational units of the EL (i.e., CCs and cell units).

All these datasets are available at: https://zenodo.org/record/3653180.

Regarding source code:

A GitHub repository with the code used to implement the computational model as well as the scripts to generate and manage the datasets used in this work is available from https://zenodo.org/record/3374889.

## Author Contributions

DD: conceptualization, data curation, formal analysis, investigation, methodology, software, validation, visualization, writing–original draft, and writing–review and editing. SR: investigation, methodology, resources, software, supervision, validation, writing–original draft, and writing–review and editing. GT: investigation, methodology, resources, software, supervision, validation, visualization, writing–original draft, and writing–review and editing. MP and AW: conceptualization, formal analysis, investigation, methodology, writing–original draft, and writing–review and editing. BZ: conceptualization, formal analysis, funding acquisition, investigation, methodology, project administration, resources, supervision, writing–original draft, and writing–review and editing.

### Conflict of Interest

The authors declare that the research was conducted in the absence of any commercial or financial relationships that could be construed as a potential conflict of interest.
